# Non-thermal hydrogen atoms in the terrestrial upper thermosphere

**DOI:** 10.1038/ncomms13655

**Published:** 2016-12-06

**Authors:** Jianqi Qin, Lara Waldrop

**Affiliations:** 1 Department of Electrical and Computer Engineering, University of Illinois at Urbana-Champaign, Urbana, Illinois, USA

## Abstract

Model predictions of the distribution and dynamical transport of hydrogen atoms in the terrestrial atmosphere have long-standing discrepancies with ultraviolet remote sensing measurements, indicating likely deficiencies in conventional theories regarding this crucial atmospheric constituent. Here we report the existence of non-thermal hydrogen atoms that are much hotter than the ambient oxygen atoms in the upper thermosphere. Analysis of satellite measurements indicates that the upper thermospheric hydrogen temperature, more precisely the mean kinetic energy of the atomic hydrogen population, increases significantly with declining solar activity, contrary to contemporary understanding of thermospheric behaviour. The existence of hot hydrogen atoms in the upper thermosphere, which is the key to reconciling model predictions and observations, is likely a consequence of low atomic oxygen density leading to incomplete collisional thermalization of the hydrogen population following its kinetic energization through interactions with hot atomic or ionized constituents in the ionosphere, plasmasphere or magnetosphere.

## Abstract (web summary)

Model predictions of atomic hydrogen behaviour in the terrestrial atmosphere have long-standing discrepancies with geocoronal observations. Here, using satellite measurements, Qin and Waldrop note the existence of hot hydrogen atoms in the upper thermosphere, reconciling observational modelling differences.

Atomic hydrogen, as the dominant neutral constituent in the upper layer of the terrestrial atmosphere, is of critical importance for many disparate aspects of aeronomy and heliophysics, such as atmospheric chemistry and energetics, ion–neutral coupling and magnetospheric energy dissipation following geomagnetic storms^1^,^2^. Moreover, as the lightest neutral species in the atmosphere, atomic hydrogen requires the lowest energy to overcome the planet's gravitational force and escape into interplanetary space. The permanent loss of hydrogen atoms, with an estimated global mean escape flux of ∼10^8^ cm^−2^s^−1^, has a significant impact on long-term atmospheric evolution^3^. Ever since the introduction of the basic concepts of atmospheric evaporation in the pioneering works of Jeans^4^ and Spitzer^5^, decades of theoretical, modelling and observational efforts have been dedicated to understanding the distribution and dynamical transport of hydrogen atoms in the thermosphere (from ∼90 to 500 km altitude) and the exosphere (above ∼500 km altitude), a region collectively known as the geocorona.

A rigorous theoretical understanding of the hydrogen geocorona has been a challenging geophysical problem that requires expertise in a wide range of disciplines, such as statistical mechanics, fluid mechanics, plasma physics, atmospheric chemistry and collision theory^1^,^3^. The complexity of the problem largely stems from the existence of a transition region between the nearly collisionless exosphere and the underlying collision-dominated thermosphere, which can be studied using neither hydrodynamic models nor collisionless kinetic models^3^,^6^. Moreover, the theoretical formulation is further complicated by the presence of non-thermal processes in the geocorona, where energetic hydrogen atoms of ∼1 eV up to ∼1 MeV can be produced by interactions such as charge exchange of the relatively cold (∼1,000 K or equivalently ∼0.1 eV) hydrogen atoms with hot protons, H^+^, in the plasmasphere^7^,^8^ and with energetic ions trapped in the magnetosphere^2^,^9^.

In the classic work of Chamberlain published in 1963, an analytic solution to the Boltzmann equation was derived for the exospheric hydrogen distribution, assuming a collisionless exosphere that has a discrete transition at the nominal exobase near ∼500 km altitude with the underlying thermosphere, along with a Maxwellian velocity distribution of the thermospheric hydrogen atoms^10^. Exospheric hydrogen atoms are partitioned into three dynamical populations, namely a ballistic component that rises from the exobase and eventually falls back, a satellite component that orbits the planet on elliptical trajectories above the exobase and an escaping component that is lost to interplanetary space along hyperbolic trajectories^10^,^11^. In subsequent studies, non-thermal processes in the plasmasphere, realized to be important or even dominant in establishing the kinetic distribution function of the satellite component and in producing energetic escaping particles, have become the main research focus to further develop exospheric theory^7^,^12^,^13^.

The Chamberlain theory, despite its unphysical collisionless assumption, has been widely used, for its relative simplicity, as the standard model in the last few decades to analyse remote sensing measurements of the hydrogen geocorona^14^,^15^,^16^,^17^, as well as to study magnetospheric energy dissipation and the resultant precipitation of energetic neutral atoms onto the thermosphere^2^,^9^. It is widely recognized that by relying on the Chamberlain model, the expected significant variation of exobase density and temperature with declining solar activity cannot be obtained from the analysis of geocoronal emission observed by the Dynamics Explorer mission^15^. Similarly, more sophisticated hydrogen density models formulated based on Monte Carlo simulation results^12^,^13^ have also been analysed with the goal of reconciling model predictions and observations^18^, although such attempts have mostly remained unsuccessful to date. A fundamental assumption of the Chamberlain theory and the Monte Carlo simulations is that the thermalization rate near and below the exobase is sufficiently high, such that the temperature of the hydrogen atoms is controlled by the ambient oxygen temperature, which decreases with declining solar activity^19^.

Here we show, on the basis of satellite remote sensing measurements and radiative transfer modelling, that the hydrogen atoms in the upper thermosphere are much hotter than the ambient oxygen atoms, especially under solar minimum conditions. In contrast to the decrease of the ambient oxygen temperature, the hydrogen temperature (that is, the mean kinetic energy of the hydrogen atoms) increases significantly with declining solar activity, likely as a consequence of the drastic decrease of atomic oxygen density leading to incomplete thermalization in the presence of non-thermal energization mechanisms.

## Results

### Observations varying with solar activity

**Figure 1:**
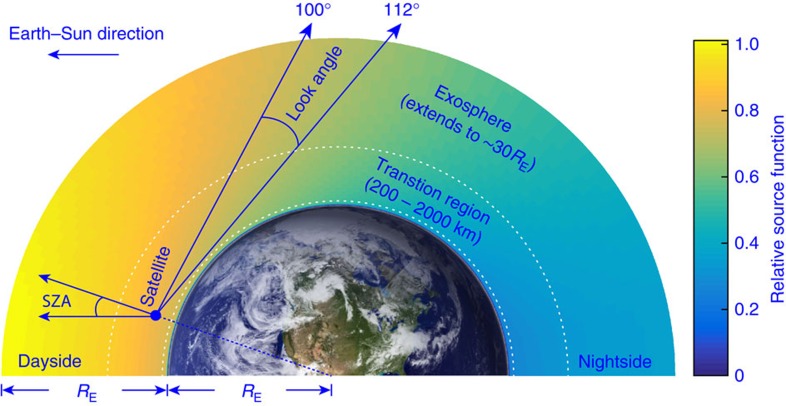
A schematic diagram of the Global Ultraviolet Imager observational geometry. The TIMED satellite is orbiting at 625 km and viewing in the anti-sunward limb direction. One example of the source function calculated using the radiative transfer model described in Methods is shown. The Earth image is taken by NASA. Note that the actual simulation domain extends up to ∼30*R*_E_, where *R*_E_ represents the Earth radius. SZA is the angle between the zenith and the Earth-sun direction.

The Global Ultraviolet Imager (GUVI) is one of the four instruments that constitute the Thermosphere Ionosphere Mesosphere Energetics and Dynamics (TIMED) spacecraft, the first mission of the NASA Solar Connections program^20^,^21^. The satellite was launched in 2002 into a nearly circular orbit at 625 km altitude with an inclination of 74° from the equator. Figure 1a shows a schematic diagram of the observational geometry of the GUVI instrument, which is viewing in the anti-sunward disk and limb direction. Observed Lyman-α (Ly_*α*_) emission at 121.6 nm is fully attributed to resonant scattering of solar Ly_*α*_ photons by hydrogen atoms in the geocorona^22^. The limb portion of the measurements, which have been binned into 32 pixels corresponding to lines-of-sight having local zenith angles of ∼100°–112°, are used in the present analysis. The tangent point altitude of those lines-of-sight range from ∼100 to 520 km, such that the GUVI measurements are most sensitive to the hydrogen density in the upper thermosphere near the nominal exobase. As described in Methods, the observed Ly_*α*_ radiances are binned and averaged both spatially, over solar zenith angle (SZA), and climatologically, over the daily solar 10.7 cm radio flux, a proxy for solar activity. With SZA bin sizes of 2° and F_10.7_ bins of 10 flux units, the number of scans included in each bin ranges from several hundreds near solar maximum, to several thousands under moderate solar activity, to more than 10^4^ during solar minimum^23^.

**Figure 2:**
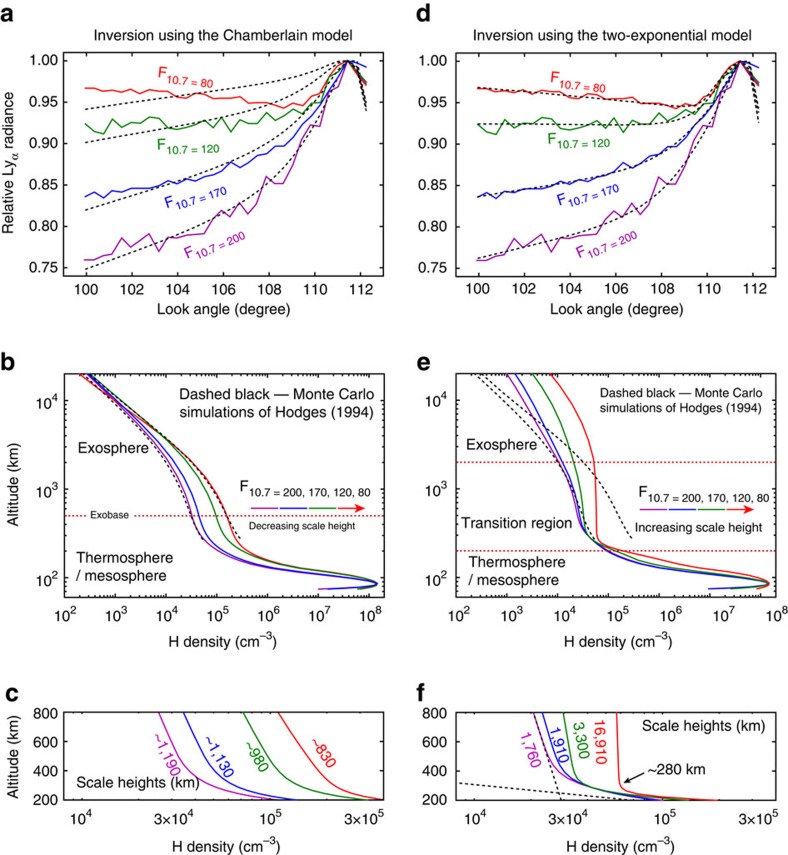
Inversion of the observed Ly_*α*_ radiances. (**a**–**c**) Inversion using the Chamberlain model as the physical constraints. (**d**–**f**) Inversion using the two-exponential model as the physical constraints. In **a**,**d** the solid-coloured lines represent the observed Ly_*α*_ radiances associated with different levels of solar activity: (red) F10.7=80; (cyan) F10.7=120; (blue) F10.7=170; and (purple) F10.7=200, and the dashed-black lines are the modelled radiances corresponding to the best-fit cases in the least squares sense. In **b**,**e**, the dashed-black lines represent the previous Monte Carlo simulation results under solar maximum and minimum conditions^13^. The solid-coloured lines are the hydrogen density profiles retrieved from the corresponding radiance data in **a**,**d** using (**b**) the Chamberlain model and (**e**) the two-exponential model as the physical constraints. (**c**,**f**) Zoom-in views of the hydrogen density profiles from **b**,**e**. The estimated scale heights in **c** are controlled by the ambient temperature specified by the NRLMSISE-00 model, which are 1,232, 1,146, 974 and 809 K, respectively. The temperature corresponding to the scale heights shown in **f** are calculated to be 2,093, 2,271, 3,923 and 20,116 K assuming hydrostatic equilibrium. It should be noted that the condition of hydrostatic equilibrium is not fulfilled in the upper thermosphere for the hydrogen population, thus the scale height–temperature relationship is not linear. The temperatures calculated based on the scale heights of **f** serve only to contrast the inferred climatological behaviour and that specified by the NRLMSISE-00 model. The two dashed-black lines in **f** represent the exponential functions of the cold and the hot populations under solar maximum condition.

Four representative cases of the observed Ly_*α*_ radiances across the dayside limb in the 20°<SZA<22° bin, associated with F_10.7_ binned in the range of 195–205, 165–175, 115–125 and 75–85, are shown in Fig. 2a,d. The radiances are normalized to the peak radiance of each case, since the hydrogen density can be quantified from the shape of the Ly_*α*_ radiances across the limb without requiring knowledge of the absolute instrumental calibration or the solar Ly_*α*_ flux at line centre, which linearly scale the measurements^16^,^23^. The four cases, labelled F_10.7_=200, 170, 120 and 80, indicate a clear trend of the Ly_*α*_ radiances varying with solar activity. With the decrease of F_10.7_ from 200 (solar maximum) to 80 (solar minimum), the relative Ly_*α*_ radiances decrease more slowly or even increase with decreasing look angle. We emphasize that this trend has profound implications on the distribution and dynamical transport of the hydrogen atoms, which likely depend more significantly on ion–neutral coupling in the terrestrial atmosphere than previously expected.

To understand the implications, it is insightful to first consider a simplified problem where individual Ly_*α*_ photons originating from the sun experience only a single scattering in the atmosphere before being measured by GUVI. In such a case, the Ly_*α*_ radiances observed at smaller look angles are associated with scattering events occurring at higher altitudes, thus larger relative Ly_*α*_ radiances observed at small look angles indicate a slower decrease of the atomic hydrogen density with increasing altitude. Further, since the GUVI measurements are most sensitive to the atomic hydrogen density in the upper thermosphere, the observed solar cycle climatology would imply that the density scale height near the exobase should increase with declining solar activity. A larger scale height indicates a higher temperature, or more precisely, a larger mean kinetic energy of the hydrogen population. Hence, the hydrogen temperature should increase with declining solar activity, in direct contrast to the fundamental assumptions of existing geocoronal theories.

The above reasoning suggests the existence of hydrogen atoms that are much hotter than the ambient oxygen atoms in the upper thermosphere, where a cold-to-hot transition of the hydrogen temperature apparently occurs with increasing altitude, since the hydrogen atoms are completely thermalized in the lower thermosphere due to exponentially larger oxygen density there. However, the single scattering assumption is not satisfied in the atmospheric region below ∼3*R*_E_, where *R*_E_ represents the Earth radius, such that the effects of multiple scattering need to be considered for a proper interpretation of the observed radiances. To prove the validity of the new findings, we use the radiative transfer model and the inverse model described in Methods to derive the unknown hydrogen density distribution from the observed Ly_*α*_ radiances.

### Cold-to-hot transition in the thermosphere

Ultraviolet remote sensing of the hydrogen geocorona is enabled by the resonant scattering processes that can transfer the solar Ly_*α*_ photons initially travelling in the Sun–Earth direction into the field-of-view of the GUVI instrument. In this study, the comprehensive radiative transfer model named *Lyao_rt*, originally developed by late J. Bishop (Bishop^22^) based on the numerical algorithm of Anderson and Hord^24^, has been thoroughly re-examined and modified to calculate the transport of solar Ly_*α*_ photons in the hydrogen geocorona. Information about the geocoronal hydrogen density that is embedded in the observed Ly_*α*_ radiances can be extracted by fitting the (radiative transfer) modelled radiances, which depend sensitively on the underlying hydrogen density distribution, to the observed radiances along the Earth's limb.

To demonstrate the long-standing discrepancies between the existing theories and observations, hydrogen density distributions parameterized based on the Chamberlain model are first used as the physical constraints to invert the observed Ly_*α*_ radiances. The solid-coloured lines shown in Fig. 2b represent the hydrogen density profiles that lead to the best-fit results shown as dashed lines in Fig. 2a. The retrieved hydrogen density profiles are similar to those obtained from the previous analysis of geocoronal observations^18^, as well as to those obtained from Monte Carlo simulations^13^. As shown in Fig. 2a, the modelled radiances fit the observations reasonably well only under solar maximum conditions. With declining solar activity, the modelled and the observed radiances become increasingly different from each other, which is not surprising since in the Chamberlain model the density scale height near the exobase decreases with declining solar activity, as shown in Fig. 2c.

The expectation of a smooth cold-to-hot transition in hydrogen temperature with increasing altitude motivates our introduction of a new model to parameterize the hydrogen density in order to properly address this incompletely thermalized regime (see Methods). Specifically, we use the sum of two-exponential functions as the physical constraints to invert the observed Ly_*α*_ radiances. The two-exponential model serves as a transition model for smoothly connecting the thermospheric and exospheric hydrogen density models developed previously by Bishop^25^,^26^. Excellent agreement is obtained under all solar conditions, as demonstrated in Fig. 2d–f, indicating that an extended, rather than a discrete, cold-to-hot transition is indeed the key to reconciling model predictions and observations.

An important feature shown in Fig. 2e is that the retrieved hydrogen density in the exosphere is significantly larger than that predicted by either the Chamberlain model or the Monte Carlo simulations, owing to the existence of a large number of satellite atoms. This finding is consistent with a recent analysis of Ly_*α*_ data acquired by NASA's Two Wide-Angle Imaging Neutral-Atom Spectrometers (TWINS) satellites, which shows that the derived hydrogen density in the altitude range from 3*R*_E_ to 8*R*_E_ decreases much more slowly with increasing altitude relative to that predicted by Monte Carlo simulations^27^. Another important feature is that the hydrogen density scale height, and thus the hydrogen temperature in the upper thermosphere, increases with declining solar activity, indicating that the upper thermosphere is not a completely thermalized regime. The cold-to-hot transition of the hydrogen temperature occurs near 440 km altitude under solar maximum conditions and near 280 km altitude under solar minimum conditions.

### Implications for geocoronal physics

**Figure 3:**
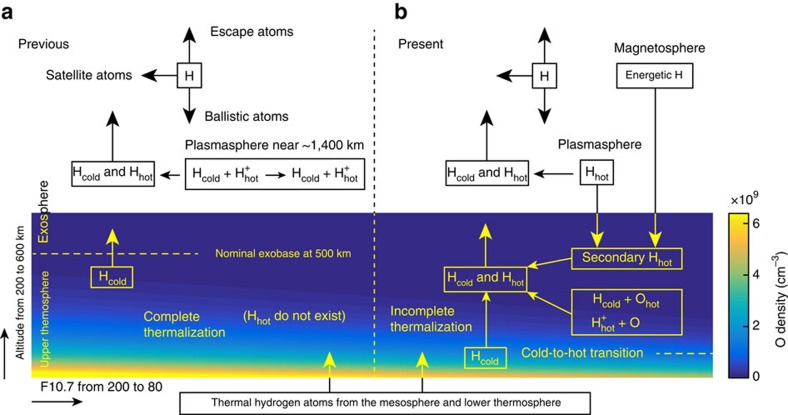
The previous geocorona theory and the new physics implied in this study. (**a**) Only cold hydrogen atoms, with a Maxwellian kinetic distribution determined by the ambient oxygen temperature, are present in the upper thermosphere, since complete thermalization is assumed. (**b**) Incomplete thermalization, due to low oxygen density especially under solar minimum condition, allows the presence of hot hydrogen atoms in the upper thermosphere. Variation of the atomic oxygen density in the upper thermosphere with solar activity is calculated using the NRLMSISE-00 model^19^. We emphasize that in the present work the cold atoms refer to those atoms that diffuse upward from the lower thermosphere (that is, the thermal atoms), and the hot atoms are the ones that are kinetically energized through processes such as charge exchange and momentum transfer.

The atmospheric region near and below the nominal exobase, namely the upper thermosphere, has long been envisioned as a fluid regime in which collisional thermalization is sufficiently rapid for the hydrogen temperature to be controlled by the ambient oxygen temperature, such that the kinetic distribution of the hydrogen atoms only slightly departs from a Maxwellian due to the escape of the high-energy portion of the thermal atoms^1^,^13^. Figure 3a depicts this historical understanding of the dynamical transport of terrestrial hydrogen atoms, showing only the most relevant processes to this study. An implicit assumption in existing geocoronal theories is that the upper thermosphere serves purely as a sink for the energy, if any, deposited by energetic hydrogen atoms from the plasmasphere and magnetosphere. In other words, no partial energy of the precipitating particles is transferred back to the exosphere by the upward flux of the ballistic and escaping hydrogen atoms. While previous studies have emphasized that the exosphere is not truly collisionless, the present analysis demonstrates that the upper thermosphere should not be considered a purely collision-dominated, and thus completely thermalized, regime either.

The existence of hot hydrogen atoms in the upper thermosphere is likely a consequence of incomplete thermalization due to insufficient atomic oxygen density following the non-thermal production of hot hydrogen atoms. Based on the previous studies of non-thermal processes in the geocorona, four possible source mechanisms of the hot hydrogen atoms are depicted in Fig. 3b and discussed as follows. One possible local source mechanism is the interaction of the thermal hydrogen atoms with superthermal oxygen atoms produced by photodissociation of O_2_ or dissociative recombination of 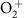 and NO^+^ near the nominal exobase^28^. Model estimation shows that at altitudes near 550 km for low solar activity and quiet geomagnetic conditions, the hot oxygen population has a temperature of ∼4,000–5,000 K (∼0.34–0.43 eV) and a number density of ∼10^5^ cm^−3^. Kinetic energization by collisions with hot oxygen has been demonstrated to be important for the escape of hydrogen from Mars and Venus^1^,^3^, but this process has not been thoroughly investigated for the terrestrial atmosphere. Another possible local source mechanism for generating hot hydrogen atoms is charge exchange between the hot ionospheric/plasmaspheric protons of ∼2,000–10,000 K (∼0.17–0.86 eV) and thermospheric oxygen atoms^3^. Hot hydrogen atoms of ionospheric/plasmaspheric origin follow ballistic trajectories and, if moving upwards, enhance the escape rate and the satellite populations, and, if moving downwards, deposit energy to the upper thermosphere.

Similarly, precipitation of energetic hydrogen atoms from the magnetosphere is likely to be another source of hot hydrogen atoms in the upper thermosphere. These precipitating atoms, which are produced by charge exchange of energetic ions trapped in the ring current and the radiation belt with the relatively cold exospheric hydrogen atoms, have extremely high energies in the range of ∼1 keV to ∼1 MeV (ref. 2). Such energetic hydrogen atoms can locally produce secondary non-thermal hydrogen atoms following their precipitation into the upper thermosphere, and the cascade heating effect can significantly enhance the thermospheric hydrogen temperature. Some of the deposited energy from the plasmasphere and the magnetosphere can be carried back to the exosphere by the upward flux of the hydrogen atoms, leading to large satellite populations and an enhanced escape rate. There is some evidence indicating a significant influence of energetic neutral particle precipitation from the ring current on the dayglow at low and mid-latitudes (<30° geomagnetic latitude)^29^,^30^. Those works show that the low-latitude dayglow emissions, including the OII 83.4, OI 98.9 and 130.4 nm, as well as the H-Ly_*α*_ emissions observed from the STP 78–1 satellite at 600 km altitude, are brightened during geomagnetically active times compared with quiet times. Moreover, analysis of TWINS observations shows that five geomagnetic storms that occurred in 2011 are accompanied by abrupt, temporary increases in the number of hydrogen atoms in the spherical shell between geocentric distances of 3–8*R*_E_, likely due to the coupling effects that exist between the exosphere and plasmasphere/magnetosphere^31^.

Regardless of their origins, the existence of a significant population of non-thermal hydrogen atoms in the upper thermosphere has a profound impact on the distribution and dynamical transport of the hydrogen atoms throughout the terrestrial atmosphere, manifesting in the retrieved hydrogen density profiles as large scale heights near the nominal exobase and large satellite populations in the exosphere. Accurate knowledge of the atmospheric hydrogen distribution is vital for numerous investigations into the chemistry, energetics, and coupling of the atmosphere, ionosphere, plasmasphere and magnetosphere. The new physics reported in this study suggests that the influence of ion–neutral coupling between the atmosphere, plasmasphere and magnetosphere on geocoronal structure and kinematics has been significantly underestimated for decades. These results provide essential knowledge for advancing development of geocoronal theory and also reveal the geocorona to be a useful arena for the study of rarified gas dynamics.

## Methods

### The GUVI data processing algorithm

Detailed information about the GUVI instrument and the data processing algorithm used in this work has been documented previously^23^ and is briefly reviewed as follows. The 11.78° field-of-view of the GUVI instrument is mapped into 14 spatial pixels along the spacecraft orbital track and 160 spectral bins spanning 115–180 nm. Multispectral images spanning the Earth's full disk to the anti-sunward limb are generated by sweeping the field-of-view from horizon to horizon perpendicular to the spacecraft motion using a scan mirror. The limb scanning portion of the image is binned into 32 pixels, corresponding to lines-of-sight having local zenith angles of ∼100°–112°. The measured spectrum is binned by an onboard detector processor to yield radiance in five distinct wavelength intervals, including H Ly_*α*_ at 121.6 nm, in each of the 14 along-track and 32 cross-track spatial bins of the limb images. Averaging of the 14 individual limb scans which comprise a GUVI limb image is performed in order to limit the noise associated with each measurement, yielding more than 5.8 × 10^6^ individual limb scans acquired from March 2002, near solar maximum, through July 2007, well into a deep solar minimum. Climatological dependencies are investigated by binning and averaging Ly_*α*_ limb scan radiances, with restriction to dayside scanning data within 45° of the geographic equator to avoid contamination by auroral emission features. The solar cycle trends are investigated by binning and averaging the radiance data in terms of the daily 10.7 cm radio flux index, F_10.7_, during the scan and in terms of the SZA of the highest-altitude tangent point for each scan. Quiet geomagnetic conditions are ensured by omitting scans having the 3 h or the daily averaged Ap index of more than 20.

### The radiative transfer model

In radiative transfer theory, the spatial variable commonly used for describing the scattering processes is the optical depth 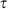 at the centre of the planetary line, given by 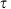
=*σ*_0_*N*, where *σ*_0_ is the line centre cross-section and *N* is the column density of the scatterers along a given optical path through the medium. An atmosphere is described as optically thin if the probability of a scattered photon encountering another atom before escaping the medium is small^24^. In the radiative transfer equation, the basic physical quantity is the source function *S*(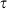), defined as the volume emission rate divided by the extinction coefficient (that is, divided by the probability per unit length of photons being scattered out of the radiation beam at location 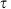). The solution is expressed in an integral form:


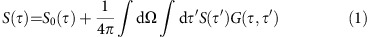


where *G*(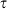, 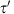) is the Holstein G-function defined as the probability that a photon emitted in d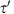 at 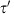 will be resonantly absorbed in d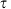 at 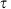, *S*_0_ is the single scattering source function of the solar Ly_*α*_ photons and the second term is the contribution from multiple scattering. Once *S*(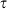) is evaluated, the radiances *I*(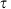) are given by the line-of-sight integral


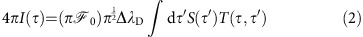


where 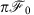 is the incident solar photon flux at line centre, *λ*_D_ is the Doppler width of the emission line and *T*(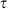, 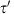) is the transmission function, defined as the probability that a photon emitted at 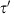 will traverse optical depth |
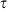
−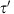
| without being scattered or absorbed. For more details about the physics in the radiative transfer theory, one can refer to recent works on the radiative transfer modelling of the OI 135.6 nm emission in the night-time ionosphere^32^,^33^.

The GUVI data analysis presented in this study is based on a spherically symmetric, non-isothermal radiative transfer model, known as *Lyao_rt*, that was developed explicitly for interpretation of ultraviolet photon scattering by atomic hydrogen in the terrestrial atmosphere by Bishop^22^. The *Lyao_rt* model assumes complete frequency redistribution and uses an anisotropic scattering function tabulated by Chamberlain^34^. Technical details of the original *Lyao_rt* code have been presented previously by Bishop^22^. Here we focus on the modifications that have been made during the re-examination of the code. In the original *Lyao_rt* code, the simulation domain is discretized exactly following the numerical scheme developed by Anderson and Hord^24^, in which the optical depth is calculated assuming constant number density within each zone to evaluate of the transmission function *T*(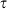, 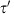). In the analysis presented here we have modified the algorithm such that the number density in each zone is assumed to vary exponentially in the vertical direction, and the integration in each zone along a given path is evaluated using Gaussian quadrature. The resulting source function has up to ∼30% difference relative to that calculated using the original model. Numerical tests have shown that the original source function approaches numerical equivalence with that of the modified model when spatial resolution is increased, indicating that the modified version is more accurate.

### The inverse model

Quantification of the hydrogen density field based on ultraviolet remote sensing of the geocorona is a typical nonlinear inverse problem with the goal of estimating unknown density from integrated line-of-sight measurements. With an orbit at 625 km altitude and a small field-of-view of ∼12°, the GUVI instrument is most sensitive to the hydrogen density in the transition region separating the thermosphere and exosphere due to the optically thick conditions at those altitudes. Since substantial hydrogen density can extend beyond ∼20*R*_E_ (ref. 13), the inverse problem is essentially an underdetermined nonlinear least squares problem, which requires the enforcement of additional constraints to guarantee solution uniqueness. This is fulfilled by incorporating physically motivated, parameterized hydrogen density distribution into the radiative transfer model. The inverse problem is solved by determining the optimal parameters of the hydrogen density profile leading to the best-fit of the (radiative transfer) modelled radiances to the observed radiances. Two distinct hydrogen density parameterizations, distinguished primarily by their treatment of the transition region between the thermosphere and exosphere, are considered here for comparison. The first parametrization exemplifies the classic approach, whereby the transition is unphysically modelled as a discrete exobase, while the second introduces a novel functional form for smoothly connecting the thermospheric and exospheric hydrogen density models developed previously by Bishop^25^,^26^. Both models are described in more detail below.

In the first hydrogen density model, exospheric density is parameterized based on the standard Chamberlain model^10^ and is extended down into the thermosphere and mesosphere as the solution of a diffusion equation combined with a Chapman profile as proposed by Bishop^26^. This density model depends on relatively few physically motivated, salient parameters, namely: the exobase density, *n*_c_; the exobase temperature, *T*_c_; the satellite critical radius, *r*_cs_; the thermospheric upward diffusive flux, *ϕ*; and the mesospheric peak density, *n*_peak_, at ∼85 km altitude. Modelling tests indicate that thermospheric Ly_*α*_ measurements are not sensitive to *r*_cs_ and *n*_peak_, which are fixed to be 2.5*R*_E_ and 1.5 × 10^8^ cm^−3^, respectively^10^,^26^. The exobase temperature, *T*_c_, is assumed to be identical to the ambient oxygen temperature, which in this study is specified by the widely used NRLMSISE-00 climatological model^19^. Thus, the two free parameters governing the hydrogen density profiles are the exobase density, *n*_c_, and the upward diffusive flux, *ϕ*, in the thermosphere.

An alternative hydrogen density parameterization that has been used to invert observed Ly_*α*_ radiances is the modified Chamberlain model developed by Bishop^25^, with extension to the thermosphere and mesosphere using the same parameterization technique mentioned above^23^,^26^. The modified Chamberlain model accounts for the effects of solar radiation pressure, and more importantly, the effects of charge exchange processes in the plasmasphere, by introducing two additional free parameters, namely the effective exobase density, *n*_s_, and the effective exobase temperature, *T*_s_, for the satellite populations. However, because this model maintains a discrete transition between the thermosphere and exosphere, the hydrogen density profiles reconstructed from GUVI data exhibit an unphysical structure at the nominal exobase (specifically a discontinuity in the density gradient) and the radiative transfer model cannot reproduce the observed minimum in the relative radiance near the 109° look angle^23^. Parameterization of the satellite population using an effective exobase temperature, on one hand, leads to a more accurate description of the hydrogen density in the exosphere, but on the other hand, allows an abrupt change of the hydrogen temperature from the ambient thermospheric temperature to the effective temperature, resulting in two significantly different scale heights above and below the exobase. To overcome the unphysical assumption of a discrete transition, we introduce a new parameterization that takes the form of the sum of two-exponential functions in the transition region from *h*=200 to 2,000 km altitude


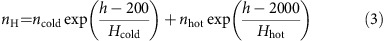


This novel functional form explicitly accounts for a smooth transition between two distinct Maxwellian populations and leads to excellent agreement between the model predictions and observations under all solar conditions, as demonstrated in Fig. 2d. The free parameters in this hydrogen density model are: the exobase density, *n*_c_; the thermospheric upward diffusive flux, *ϕ*; the effective exobase density, *n*_s_; the effective exobase temperature, *T*_s_; and the two scale heights, *H*_cold_ and *H*_hot_. The quantities *n*_cold_ and *n*_hot_ are determined by equating the densities at 200 and 2,000 km altitudes to those given by the Bishop thermospheric model^26^ and the modified Chamberlain exospheric model^22^, respectively.

For brevity, the two hydrogen density models are referred to as the Chamberlain model and the two-exponential model, respectively, in this work. The background atmosphere, such as the atomic oxygen density and the ambient temperature, are also required for the radiative transfer modelling of the Ly_*α*_ photons in the terrestrial atmosphere. In the present study, those quantities are specified using the NRLMSISE-00 model^19^, corresponding to a dayside (SZA=18°) location on the geocentric solar ecliptic equator at geographic location (−5°, 0°) during the vernal equinox at 11:00 UT (∼14.25 h local time)^23^. The optimal parameters of the hydrogen density profiles leading to the best-fits of the (radiative transfer) modelled and the observed radiances are obtained through an iterative process using the Gauss–Newton method^35^.

### Data availability

The data that support the findings of this study are available from the corresponding author upon reasonable request.

## Additional information

**How to cite this article:** Qin, J. & Waldrop, L. Non-thermal hydrogen atoms in the terrestrial upper thermosphere. *Nat. Commun.* **7,** 13655 doi: 10.1038/ncomms13655 (2016).

**Publisher's note**: Springer Nature remains neutral with regard to jurisdictional claims in published maps and institutional affiliations.
